# The Efficacy of Inositol and N-Acetyl Cysteine Administration (Ovaric HP) in Improving the Ovarian Function in Infertile Women with PCOS with or without Insulin Resistance

**DOI:** 10.1155/2014/141020

**Published:** 2014-04-30

**Authors:** Angela Sacchinelli, Roberta Venturella, Daniela Lico, Annalisa Di Cello, Antonella Lucia, Erika Rania, Roberto Cirillo, Fulvio Zullo

**Affiliations:** Unit of Obstetrics and Gynaecology, Department of Experimental and Clinical Medicine, “Magna Graecia” University, Viale Europa, Germaneto, 88100 Catanzaro, Italy

## Abstract

*Objective.* Substances such as inositol and N-acetylcysteine (NAC) have been recently shown to be effective in treatment of PCOS patients. The aim of this prospective trial is to evaluate the efficacy of NAC + Inositol + folic acid on ovulation rate and menstrual regularity in PCOS patients with and without insulin resistance.* Methods.* Among the 91 PCOS patients treated with NAC + Inositol + folic, insulin resistance was present in 44 subjects (A) and absent in 47 (B). The primary endpoint was the ovulation rate/year, determined by menstrual diary, serum progesterone performed between 21° and 24° days, ultrasound findings of growth follicular or luteal cysts, and luteal ratio. HOMA-index assessment after 6 and 12 months of treatment was evaluated as secondary endpoint.* Results.* In both groups there was a significant increase in ovulation rate and no significant differences were found in the primary outcome between two groups. In group A, a significant reduction of HOMA-index was observed.* Conclusions.* The association NAC + Inositol + folic, regardless of insulin-resistance state, seems to improve ovarian function in PCOS patients. Therefore, inositol and NAC may have additional noninsulin-related mechanisms of action that allow achieving benefits also in those patients with negative HOMA-index.

## 1. Introduction

Polycystic ovary syndrome (PCOS) is characterized by chronic oligo/anovulation and hyperandrogenism (clinical and/or biochemical) and is the most common cause of anovulatory infertility and menstrual irregularities [1]. PCOS affects 5–10% of women of reproductive age and therefore is the most common endocrine-metabolic premenopausal disorder [2].

Its pathogenesis is not yet entirely clear, although in recent years many researchers have focused their attention on the role of glucose tolerance and insulin resistance, placing these mechanisms among the most important responsible of this complex syndrome. In fact, insulin resistance is found in 30–40% of women with PCOS [3], and it is characterized by resistance to the effects of insulin and in uptake in the metabolism of glucose with subsequent reduction of body's ability to eliminate glucose from the bloodstream. This condition, also determining an increased risk of type 2 diabetes mellitus, dyslipidemia, cardiovascular disease, and cancer, causes a significant impact on women fertility [4]. Although the pathogenetic mechanism is not yet fully understood, several hypotheses have been formulated on how insulin resistance may lead to a reduction in the female reproductive chances.

On the one hand, some authors believe that the hyperinsulinemia, secondary to insulin resistance, stimulates the secretion of both ovarian and adrenal androgen and suppresses the synthesis of sex hormone binding globulin (SHBG) by the liver, causing a consequent increase of biologically active androgens in the circulation. The excessive ovarian androgen production would result in early follicular atresia and anovulation, along with all the other typical clinical manifestations of hyperandrogenism, including hirsutism and acne [5].

Other authors believe that the alteration of intracellular signaling pathways in response to insulin (in which the inositol plays an important role as a second messenger) is crucial in the development of this syndrome [4]. The measurement of insulin resistance is currently achieved using the HOMA-IR (Homeostasis Model of Assessment-Insulin Resistance), calculated by multiplying the fasting plasma insulin (expressed in mU/L) for the levels of fasting plasma glucose (expressed in mmol/L) and dividing the result by the constant 22.5.

Myoinositol is an isoform of inositol, belonging to vitamin B complex, with insulin-like action in vivo and functions as a mediator of insulin. It plays an important role in cellular morphogenesis and cytogenicity, in the synthesis of lipids, and in the structure of cell membranes and cell growth. Data in the literature have shown that in some tissues there is a correlation between insulin resistance and reduction of myoinositol [6] and some authors suggest that a deficiency in the availability or use of myoinositol may be responsible for a mechanism of insulin resistance, with consequent effects on ovarian function [6, 7]. In particular, it seems that in the theca cells of PCOS patients sensitive to insulin, the imbalance of inositol goes in the opposite direction to that observed in cells of insulin-resistant women. Supplementation of inositol, therefore, would seem to improve the ovarian response to gonadotropin-induced compensatory hyperinsulinemia, in addition to improving oocyte quality [6].

For some time the attention of the recent scientific literature has shifted to a new molecule: the N-acetyl- cysteine (NAC). Recent data have shown that NAC, a mucolytic drug acting as insulin sensitizer, represents an effective and safe strategy in the treatment of PCOS patients [8, 9]. This molecule appears to exert its beneficial effect both by increasing the insulin secretion by the beta cells of the pancreas and by inducing an increased sensitivity to the organism itself. In particular, some data show that a moderate increase in plasma thiols improves the consumption of glucose during the hyperglycemic clamp [9]. Other studies suggest a role of  NAC in the regulation of the insulin receptor in human erythrocytes [10]. It has been shown, in fact, that high doses of NAC increase the cellular levels of reduced glutathione (GSH), a powerful antioxidant that seems to exert a certain influence on activity of the insulin receptor in vivo [11].

In vitro data also show that cysteine and its analogs are able to potentiate insulin secretion induced by glucose. This observation suggests the presence of a specific molecular portion in the cysteine that is necessary for its insulinotropic effect [11].

Observing the molecular structure of NAC (N-acetyl-cysteine) highlighted the presence of a disulfide bridge that should facilitate the processing of proinsulin into insulin, a critical step to reduce insulin resistance.

In humans, oral administration of  NAC has also been proposed for the prevention of endothelial damage by oxidizing agents in adult insulin-dependent diabetics [12]. Therefore, administration of NAC in PCOS patients could have important long-term effects on insulin secretion, on metabolism, and on peripheral insulin sensitivity.

On the basis of the observed data, we conducted a prospective study whose objective was to compare the efficacy in terms of ovulation induction and normalization of menstrual cycles of combined treatment with NAC + Inositol + folic acid (Ovaric HP) in insulin-resistant patients with PCOS compared with the effects of the same drug in non-insulin-resistant patients with PCOS.

## 2. Materials and Methods

The present study was conducted at the Department of Obstetrics and Gynecology of the University Magna Graecia of Catanzaro. All the procedures of the Protocol have been carried out in accordance with the rules of the Helsinki Declaration on human experimentation and the Good Clinical Practice. The purpose of the study was carefully explained to each patient and informed consent was obtained and signed by each of them prior to study entry.

The aim of this study was to evaluate the effectiveness of the new combined treatment (Inositol + folic acid + NAC) in restoring menstrual regularity and ovarian cyclicity regardless of the status of insulin resistance. The primary endpoint was the ovulation rate per year. The restoration of a spontaneous ovulatory cycle was determined by a menstrual diary kept by the patient on a monthly basis as well as through an assessment of serum progesterone (P) performed between 21° and 24° days and a transvaginal ultrasound to document the presence of follicular growth or luteal cysts. A serum P > 2.5 ng/mL was considered indicative of spontaneous ovulation. The ovulation rate was also calculated using the ratio between the weeks of luteal phase and the weeks of observation (luteal ratio), so that a woman with normal menstrual rhythm would show two luteal weeks in four weeks of observation, obtaining a ratio of 0.5, expressed as luteal ratio of 50%. The secondary endpoint was to evaluate the HOMA index at 6 and 12 months.

The diagnosis of PCOS was made on the first visit by ultrasound evaluation, clinical biochemistry, and in accordance with the criteria of the Consensus Conference in Rotterdam (ESHRE/ASRM, Sponsored PCOS Consensus Workshop Group, 2003). In particular, the diagnosis was made in the presence of at least two of the following criteria: (1) clinical or biochemical hyperandrogenism (Ferriman-Gallwey score ≥ 8 (Ferriman 1961) or serum testosterone levels > 2 standard deviation (SD) of the average values of reference), (2) chronic oligoanovulation, (3) PCO morphology at the ultrasonography examination. The ultrasonographic ovarian morphology was assessed by a single experienced operator.

Exclusion criteria were considered: age below 18 and over 35 years, obesity (BMI > 30 kg/m^2^), precancerous lesions or cancer, major medical conditions (including hypertension and/or diabetes mellitus), or other comorbidities; hypothyroidism, hyperprolactinemia, Cushing's syndrome, congenital adrenal hyperplasia, nonclassical clotting disorders, autoimmune diseases, cigarette smoking, use of drugs/alcohol; organic pelvic pathology, uterine malformations, previous pelvic surgery, patients do not compliant the study protocol; concurrent or previous (within the last six months) use of hormonal drugs, antidiabetic and antiobesity drugs, multivitamins, and folic acid. It has been considered an elective criterion for exclusion taking fertility drugs in the period prior to the study.

Moreover, women were excluded from the study if they had intention of starting a diet or a specific program of physical activity.

The enrolled patients were divided into the following treatment groups: (a) study group: patients diagnosed with PCOS and insulin resistance (HOMA index > 2.5); (b) control group: patients diagnosed with PCOS and insulin sensitive (HOMA index ≤ 2.5).

All patients of both groups have been treated with folic acid and inositol 2 g + 200 g + NAC (Ovaric HP, Just Pharma, Rome, Italy) for two times a day for 12 months, during which they were asked to record the presence and characteristics of menstrual bleeding.

For all patients, baseline characteristics such as age, anthropometric evaluation (body mass index (BMI)), hormonal status (follicle-stimulating hormone (FSH), 17-*β* estradiol (E2), progesterone (P) and testosterone (T)), and metabolic parameters (fasting glucose and insulin) were collected. At baseline, blood samples were obtained after a 12-hour overnight fast in the early proliferative phase (between first and third day) of a cycle induced by an intramuscular injection of progesterone. In all patients, during the same cycle, plasma assays of progesterone between 21° and 24° days of the cycle were also performed. Serum P level < 2.5 ng/mL was considered indicative of ovulatory failure. The HOMA-IR was calculated by multiplying the fasting plasma insulin expressed in mU/L for the levels of fasting plasma glucose expressed in mmol/L and dividing by the constant 22.5. Patients were defined insulin resistant if HOMA-IR > 2.2.

## 3. Statistical Analysis

The sample size was calculated on the basis of the published results of previous studies related to changes in rates of ovulation in insulin-resistant patients treated with inositol. For our study it was necessary to recruit 35 patients per group to highlight a significant difference in rates of spontaneous menstruation before and after treatment, with an alpha error of 0.05 and a power of 90%. Considered a dropout rate of 15%, at least 81 patients had to be enrolled.

Statistical analysis was performed using SPSS 9.0 (SPSS, Inc., Chicago, IL). The normal distribution of continuous variables was assessed using the Kolmogorov-Smirnov test. Since continuous variables showed a normal distribution, data were expressed as mean and standard deviation (SD). Continuous variables were analyzed using the Student's *t*-test for independent samples.

## 4. Results

From January to November 2012, 91 PCOS women were enrolled, 44 belonging to group A and 47 to group B. As detailed in Table 1, at baseline there was no significant difference between the two groups for age, BMI, FSH, E2, P, T.

The levels of insulin and glucose, and consequently the HOMA index, were higher in group A than in group B and this difference was statistically significant (*P* < 0.05). At baseline, the levels of P, measured between the 21st and the 24th day of the induced cycle, were similar between the two groups, as well as the number of cycles/year.

Concerning the ovulation rate, 6 months after the beginning of the study, in 5 of 44 patients with positive HOMA index and in 7 of 47 women with negative HOMA index, ovulatory cycles were equally observed (*P* = 0.449) This result was maintained even after 12 months of treatment (Table 2), with an ovulation failure in 4 patients in the first group and 5 in the second one (*P* = 0.859).

Group A showed an ovulation rate (in terms of luteal ratio) slightly greater than group B at 6 and 12 months of treatment; this difference, however, was not statistically significant between the two groups.

The serum levels of P in the luteal phase, assessed after 6 and 12 months of treatment, were similar in the two groups, demonstrating that the majority of ovulatory cycles showed a normal endocrine profile in both groups (Table 2).

On the basis of the menstrual diaries kept by the patients before and after the beginning of treatment, in both groups a significantly greater number of menstrual cycle after 6 and 12 months compared to baseline was found. However, this difference was not significant between the two groups (Table 2).

After 6 months, the glucose and insulin blood levels were lower in group A compared to baseline, as well as the HOMA index. This difference was statistically significant (*P* < 0.001). Similarly, in the group of HOMA positive patients, plasmatic levels of glucose and insulin have been shown to be reduced after 12 months of treatment, compared with baseline, significantly (*P* < 0.001).

## 5. Discussion

The role of insulin resistance in menstrual dysfunction in women with PCOS is already well known and documented in the literature. The importance of insulin resistance in the pathogenesis of the menstrual cycle disorders and infertility is also suggested by the observation that the administration of insulin-sensitizing drugs, such as metformin, can induce an improvement in spontaneous ovulation, promotes efficiency of ovulation-inducers drugs, and increases pregnancy rate [8].

Particularly, a recent systematic review of the current literature [9] showed that administration of inositol in women with PCOS would improve both their ovarian function and their metabolic and hormonal pattern. In fact, by restoring the physiological ovarian response to gonadotropins, inositol reduces hyperandrogenemia and restores the cyclical ovarian and menstrual cycles, so inducing, consequently, an increase in the probability of achieving a spontaneous pregnancy [9]. Santini et al. [10] have shown that approximately 88% of women obtain at least one spontaneous menstrual cycle during treatment, and among of these, 72% will maintain ovulatory cycles at followup. About 40% of women get pregnant after treatment with inositol [10].

In addition, considering the diet as a crucial factor for the maternal health [11] it is rational to assume that the administration of inositol and micronutrients in combination could have a beneficial effect on the reproductive health of women.

Some randomized controlled trials have shown that a combined treatment with NAC and clomiphene citrate (CC) is more effective than CC alone when it is used as second line in CC-resistant women [12, 13] or in those undergoing laparoscopic ovarian drilling [14].

To date, although there is evidence about the effects of individual components administered, no data in the literature are available regarding the efficacy of a combined treatment with inositol and NAC.

Therefore, we decided to conduct a study to evaluate the effectiveness of the administration of Inositol and N-acetyl cysteine (Ovaric HP) in terms of improvement of ovarian function in infertile women affected by PCOS with or without insulin resistance.

Considering our results it can be affirmed, as expected, that the use of Ovaric HP leads to an advantage in terms of improvement of ovulatory cycles in insulin-resistant patients subjected to the treatment. The interesting data is that this improvement, both in terms of ovulation rate and of luteal ratio and menstrual cycle restoration, is also evident in the group with negative HOMA index.

Our results demonstrate that the treatment with Ovaric HP is helpful in restoring ovulation in PCOS patients with oligomenorrhea. Furthermore, it is possible to assume that Inositol and NAC may have additional noninsulin related mechanisms of action that allow achieving beneficial effects also in those patients who are noninsulin resistant.

These benefits are probably due to the indirect action of NAC. This molecule, as demonstrated by some studies [14, 15], seems to be able to work in synergy in all forms of PCOS, both in those induced by insulin resistance and in cases of tissue oxidative stress.

## Figures and Tables

**Table 1:** Anthropometric and hormonal characteristics at baseline.

	Patients HOMA +	Patients HOMA −	*P*
Age (years)	29.2 ± 2.21	29.46 ± 2.95	0.633
BMI (kg/m^2^)	26.19 ± 2.17	26.72 ± 1.94	0.223
FSH (mIU/mL)	7.25 ± 1.65	7.07 ± 1.48	0.595
E2 (pg/mL)	33.94 ± 8.47	32.93 ± 6.61	0.527
P (ng/mL)	1.13 ± 0.46	1.31 ± 0.54	0.098
T (ng/mL)	6.29 ± 1.14	6.20 ± 1	0.702
Fasting glucose (mg/dL)	95.47 ± 8.22	86.85 ± 6.17	<0.05
Fasting insulin (*μ*U/mL)	15.04 ± 2.40	8.38 ± 2.35	<0.05
HOMA index	3.5 ± 0.52	1.77 ± 0.52	<0.05
P (ng/mL) [21°–24° cycle day]	1.99 ± 0.42	2.1 ± 0.47	0.245
Cycles/year	3.95 ± 0.86	4.23 ± 0.72	0.098

Data expressed as mean ± SD.

**Table:** (a)

	Patients HOMA +	Patients HOMA −	*P*
Fasting glucose (mg/dL)	89.75 ± 8.71	87.29 ± 1.31	0.146
Fasting insulin (*μ*U/mL)	7.91 ± 2.03	8.02 ± 0.82	0.728
HOMA index	1.73 ± 0.51	1.68 ± 0.23	0.541
P (ng/mL) [21°–24° cycle day]	2.85 ± 0.72	2.85 ± 0.77	0.984
Cycles/year	6.65 ± 1.27	6.44 ± 1.38	0.449
Ratio luteal phase (%)	35.22 ± 2.55	34.53 ± 2.41	0.185

**Table:** (b)

	Patients HOMA +	Patients HOMA −	*P*
Fasting glucose (mg/dL)	84.34 ± 7.43	85.63 ± 6.39	0.374
Fasting insulin (*μ*U/mL)	8.4 ± 1.33	8.48 ± 1.28	0.771
HOMA index	1.71 ± 0.46	1.72 ± 0.35	0.83
P (ng/mL) [21°–24° cycle day]	2.92 ± 0.58	2.93 ± 0.65	0.892
Cycles/year	7.63 ± 1.18	7.68 ± 1.19	0.859
Ratio luteal phase (%)	36.77 ± 2.76	35.82 ± 3.24	0.141

Data expressed as mean ± SD.
