# Corrigendum to “The Efficacy of Inositol and N-Acetyl Cysteine Administration (Ovaric HP) in Improving the Ovarian Function in Infertile Women with PCOS with or without Insulin Resistance”

**DOI:** 10.1155/2016/2026056

**Published:** 2016-08-17

**Authors:** Angela Sacchinelli, Roberta Venturella, Daniela Lico, Annalisa Di Cello, Antonella Lucia, Erika Rania, Roberto Cirillo, Fulvio Zullo

**Affiliations:** Unit of Obstetrics and Gynaecology, Department of Experimental and Clinical Medicine, “Magna Graecia” University, Viale Europa, Germaneto, 88100 Catanzaro, Italy

In the article titled “The Efficacy of Inositol and N-Acetyl Cysteine Administration (Ovaric HP) in Improving the Ovarian Function in Infertile Women with PCOS with or without Insulin Resistance,” [1] there was a typing mistake in the “Materials and Methods” section on page 3 in terms of the dosage of the product administered to patients. The correct sentence should be as follows.

All patients of both groups have been treated with folic acid 200 mcg, inositol 2000 mg, and NAC 600 mg (Ovaric HP Just Pharma, Rome, Italy) for two times a day for 12 months, during which they were asked to record the presence and characteristics of menstrual bleeding.
